# An Innovative Strategy for Oral Biofilm Control in Early Childhood Based on a Resveratrol-Cyclodextrin Nanotechnology Approach

**DOI:** 10.3390/ma14143801

**Published:** 2021-07-07

**Authors:** Giovanni Nicolao Berta, Federica Romano, Roberta Vallone, Giuliana Abbadessa, Federica Di Scipio, Patrizia Defabianis

**Affiliations:** 1 Department of Clinical and Biological Sciences, University of Turin, 10043 Orbassano, Italy; giuliana.abbadessa@unito.it (G.A.); federica.discipio@unito.it (F.D.S.); 2 Department of Surgical Sciences, C.I.R. Dental School, University of Turin, 10126 Turin, Italy; federica.romano@unito.it (F.R.); patrizia.defabianis@unito.it (P.D.); 3 Private Practice, 10100 Turin, Italy; vallone.roby8@gmail.com

**Keywords:** children, cyclodextrins, gingivitis, plaque control, resveratrol, oral hygiene

## Abstract

The purpose of this randomized controlled study was to evaluate the clinical efficacy of a novel oral spray containing resveratrol (RV) in controlling bacterial biofilm and gingival inflammation in early childhood. RV, a natural polyphenol, known for its anti-inflammatory and anti-infective activities, was included in a nanovector of 2-hydroxypropyl-beta-cyclodextrins (HPβCD) to improve its bioavailability. A total of 64 children between two and five years of age with plaque-induced gingivitis were randomly included in two equal groups. Both groups were enrolled in a mechanical plaque control program for a period of four weeks, while the test group was also instructed to use the RV-HPβCD mouthwash (in spray formulation) once daily, after toothbrushing. All children underwent three oral hygiene motivation sessions, 14 days apart, during which the full-mouth presence of bacterial plaque, gingival inflammation, dental stain and salivary pH were recorded. At two-week appointment, they also received professional plaque removal. The use of RV-based oral spray significantly reduced the amount of dental plaque and the percentage of bleeding sites and improved salivary pH compared to the control group at both two- and four-week examinations. Based on these promising results, the local delivery of RV-HPβCD via oral spray could enhance the control of dental biofilm in early childhood, when antiseptic mouthwashes are not recommended.

## 1. Introduction

Despite the great commitment of parents, oral hygiene in early childhood has proved to be inadequate in many cases, increasing the risk of dental decay in primary dentition [1]. A recent study demonstrated that caries prevalence at the age of five was significantly related to toothbrushing procedures performed less than twice a day [2]. In Italy, 20% of four-year-old children have dental carious lesions [3].

Along with caries, bacterial biofilm is an essential etiological factor in gingival inflammation [4]. Although, in early childhood, this response to plaque accumulation is clinically less evident in gingival tissues [5], about one third of five-year old children are affect by gingivitis [6]. The presence of erupting teeth may further increase the biofilm retention resulting in a more severe gingival inflammation [7]; for all these reasons supplementing normal oral hygiene with chemicals may be useful. Nonetheless, the use of antiseptics in mouthrinse formulation, even if effective in controlling plaque and gingival inflammation [8], is often associated with negative local side effects. Therefore, these formulations are not recommended in children younger than six years who do not split effectively with the risk of severe gastrointestinal and systemic adverse effects [9].

In the last decades, many natural compounds have been identified as molecules characterized by anti-inflammatory/antibacterial activity without local or systemic toxicity on mammalian tissues. Among them trans-resveratrol (RV, 3,5,4′-trihydroxy-trans-stilbene), a non-flavonoid polyphenol belonging to the class of stilbenes, is one of the most studied and promising compounds [10]. It is found in various vegetables and fruits, including grape skin and acts as a phytoalexin (a class of vegetal antibiotics) thus, protecting the plant from environmental stress or infections [10]. Recent studies have shown its ability to downregulate inflammatory biomarkers produced by epithelial cells, fibroblasts and leucocytes, including interleukin (IL)-1, IL-6, IL-17 and tumor-necrosis factor-α (TNF-α) [11] and to upregulate IL-10, an anti-inflammatory cytokine [12]. RV molecule is also an antioxidant. It stimulates cells antioxidant defense and can act as a scavenger itself of reactive oxygen species [13]. Furthermore, it has been found to be safe and well-tolerated up to 5 g/day, either as a single dose or as fraction of multiple-day dosing schedule [14].

Unfortunately, clinical applicability of RV is limited by its chemical-physical properties, mainly related to its low solubility in water and extensive systemic metabolism leading to a rapid inactivation. Indeed, these aspects affect negatively its bioavailability and efficacy [15]. Recently, cyclodextrins (CD), a natural oligosaccharide compound family, have been used to address this issue, modifying the chemical and physical properties of the guest compounds included in their ring-shaped structure [16]. Indeed, the complexation of RV with 2-hydroxypropyl-β-CD (HPβCD), a semisynthetic CD, has been proved effective in enhancing RV water solubility, bioavailability and efficacy [17,18]. A recent study in a mouse model demonstrated that the oral administration of RV-HPβCD increased two-fold the RV tissue bioavailability than when delivered into aqueous nanosuspension [19]. Furthermore, HPβCD has an excellent safety profile, resulting neither in local nor in systemic toxic effect on oral tissues after ingestion [18,20].

The aim of this randomized controlled clinical study was to evaluate the efficacy and acceptance of a novel RV-HPβCD complexation mouthrinse in oral spray formulation in controlling bacterial biofilm and gingival inflammation in early childhood.

## 2. Materials and Methods

### 2.1. Study Design

The children included in this randomized, two parallel arms clinical study were consecutively selected among those referred to the Section of Pediatric Dentistry, C.I.R. Dental School, Department of Surgical Sciences, University of Turin (Italy) from September 2019 to February 2020. The study was approved by the Institutional Ethics Committee of the “AOU Città della Salute e della Scienza”, Turin, Italy (No. approval 0012934) and was conducted according to the Helsinki Declaration. Parents/guardians of each recruited child signed informed consent.

Children of both genders were screened for enrolment according to the following criteria: (i) diagnosis of plaque-induced gingivitis with full-mouth bleeding score (FMBS) > 10% [21]; (ii) age between 2 and 5 years (inclusive); (iii) healthy conditions; (iv) full-mouth plaque score (FMPS) > 20% at the screening visit. Exclusion criteria were: (i) extensive caries; (ii) systemic diseases or conditions that could interfere with the study outcomes (e.g., diabetes mellitus, cancer, immune deficiencies, hematologic diseases); (iii) use of medications that cause gingival enlargement (e.g., cyclosporine, phenytoin); (iv) physical limitation or motor incoordination; (v) failure to comply with the spray regimen.

### 2.2. Mounthwash Formulation

RV-HPβCD complexation has already been described [18,19]. The mouthwash was obtained as follows: RV (0.318% *w*/*v*, purchased from Fagron, Bologna, Italy) and HPβCD (0.318% *w*/*v*, purchased from Roquette, Lestrem, France) were mixed with deionized water in a clean and sterile recipient by means of a dissolver operated to shake at low speed without swirling for 5 h.

Then, a strawberry liquid aroma (Nyl Laboratories, Monterotondo, Italy) was added in an amount (weight/volume) of 0.1%, together with deionized water to reach 100% *w*/*v*. The choice to use the strawberry aroma was based on the results obtained in a preliminary study where a sample of children preferred it over the flavors tested (data not shown) and on the findings from previous published reports [22,23]. The obtained suspension was filtered and then packaged in a bottle provided with a spray solubilizer. All described procedures were performed by Logidex (Turin, Italy).

### 2.3. Sample Size and Randomization

Children were distributed randomly in two balanced groups using a computer-generated table by an operator not involved into the trial. The test group underwent toothbrushing and daily supervised application of the RV-HPβCD oral spray and the control group only toothbrushing. To conceal assignment, forms with the treatment modality were put into sealed and opaque envelope and provided to a dental hygienist.

A sample size of 27 children per group was calculated to detect a minimum difference of 10% in FMPS between the groups with an alpha set at 5% and a power of 80% based on a previous study that found FMPS change of 32.0 ± 16.0% after mechanical plaque control [24]. An additional 20% patients per group were included for the study, considering potential loss during the follow-up period, giving a final sample size of 32 patients per group.

### 2.4. Clinical Procedures

A single specialist in pediatric dentistry assessed the oral status of the participants. All parents/guardians and all children aged 4 to 5 years of both groups received instruction by an experienced dental hygienist to perform the roll technique toothbrushing twice a day with a soft bristle-small head toothbrush. Oral hygiene procedures in children aged 2 to 3 years were performed by the parents, while those aged 4 to 5 years carried out the procedures by themselves, under parental supervision [25]. The manual toothbrush and fluoridated toothpaste were provided for use through the study. A separate hygienist provided the RV-HPβCD oral spray to the parents of the test group children and instructed them to use the spray once a day, in the evening, 30 min after plaque removal, for 4 weeks. The spray was applied along the gingival margin on the buccal and lingual aspects of all the erupted teeth. Children of the control group underwent only mechanical plaque removal without placebo use. After 2 weeks, children from both groups received professional plaque removal consisting in supragingival polishing; at both 2- and 4-week examinations their oral hygiene standards were recorded and oral hygiene procedures were reinforced. The compliance in the use of the spray was evaluated at each visit.

### 2.5. Clinical and Patient-Reported Outcomes

At baseline, 2-week and 4-week examinations clinical parameters were recorded by a blinded and experienced examiner using a 1-mm marked manual periodontal probe (PCPUNC 15, Hu-Friedy, Chicago, IL, USA). Plaque accumulation and gingival inflammation were assessed dichotomously (0 = absence; 1 = presence) at 6 sites per tooth and expressed as percentage of tooth surfaces harboring plaque (FMPS) and bleeding on gentle probing (FMBS) [26]. The presence and intensity of stains was measured on the facial aspects of the incisor teeth using the Lobene Stain Index [27]. Adverse effects were also recorded.

At the same assessment time points, unstimulated whole saliva samples were collected under standardized conditions (at least 2 h after meals and 1 h after tooth brushing) for the quantification of salivary pH using a chair side kit with a measuring range from 5.0 to 7.8 (Saliva Check Buffer System GC America Inc., Alsip, IL, USA).

At the end of the study, parents of test children were asked to complete a questionnaire on the acceptability of the oral spray. Furthermore, children of the test group rated their taste perception using a 3-point scale (good, intermediate, bad) presented with emoticons to improve their comprehension.

### 2.6. Statistical Analysis

Data were entered into a database and analyzed using statistical software (SPSS Statistics for Mac, v. 25.0, IBM, Chicago, IL, USA). All children had coded identification so as they could not be identified. Primary outcome variable was change in FMPS from baseline to 4-week examination. Secondary variables were changes in the other clinical parameters and in salivary pH.

Continuous data were examined for normality with the Kolmogorov-Smirnov and Shapiro-Wilk tests. To test the effect of treatment and time on quantitative variables within each experimental group the repeated measures analysis of variance or the Friedman test were used as appropriate, followed by post hoc tests to explore differences between time points. The statistical significance of the differences between the groups was evaluated using the Chi Square test for qualitative variables and unpaired *t* test or the Mann-Whitney *U*-test for quantitative variables. The Bonferroni correction was applied for multiple comparisons.

A secondary analysis was performed to explore the impact of the toothbrushing behavior (toothbrushing performed by the parents or by the child under parental supervision) on the outcomes using the analysis of variance or the Kruskal-Wallis test for between group comparison, and the repeated measures analysis of variance or the Friedman test for intragroup analysis. Differences were considered statistically significant when *p* was <0.05.

## 3. Results

### 3.1. Population

**Figure 1:**
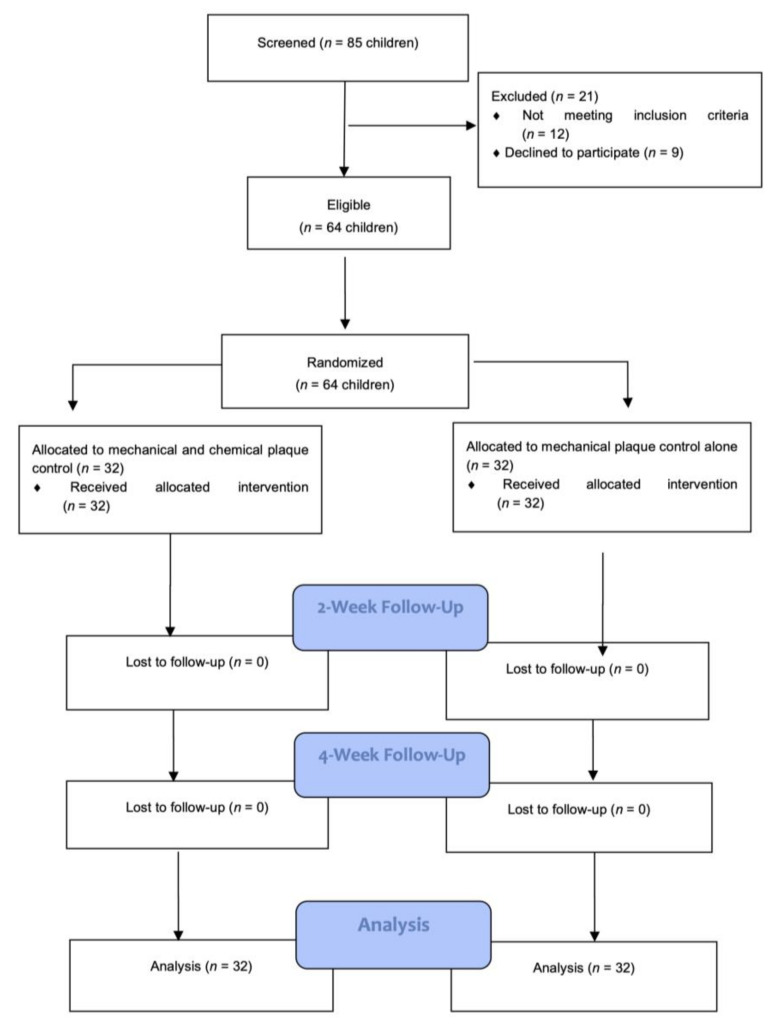
Flow chart of the study.

As shown in Figure 1, 85 children were screened for inclusion: 21 were excluded because they did not meet the inclusion criteria or parents withdrew consent. A total of 64 Caucasian children were consecutively selected for enrolment in the study; 32 subjects were assigned to the test group, and; 32 to the control group and all were scheduled for the 2-week and 4-week examinations. No data were missing for the analysis. The parents’ educational level and socioeconomic status was comparable between the two groups.

The distributions of gender and age for each group are presented in Table 1. The mean age for both groups was approximately 4 years and the numbers of males and females were nearly equal in both groups.

### 3.2. Clinical and Salivary Outcomes

The comparative analysis between test and control groups over the study period in relation to clinical parameters is shown in Table 2. The groups were balanced for FMPS and FMBS values at the beginning of the study.

Both treatments were associated with statistically significant reduction in the percentage of sites with plaque and in the degree of gingival inflammation with a decrease from baseline to 4-week follow-up (all *p* < 0.001). After both 2 and 4 weeks of daily use, the RV-HPβCD group had significantly less plaque than the control group with reduction of 40–50%. A similar effect was observed for FMBS values which decreased more in the test group at both assessment time points (*p* < 0.001).

Clinical data were stratified based on the age of the enrolled children to explore whether there was any association between the use of the RV-HPβCD spray and the toothbrushing behavior (Table 3). From the study group of 24 children aged 2 to 3 years, whose teeth were regularly brushed by their parents, 11 received the spray and 13 did not. From the 40 children aged 4 to 5 years, who performed toothbrushing under parental supervision, 21 used the spray and 19 did not. While at baseline there was no difference among the groups, at 2-week and 4-week evaluations FMPS and FMBS decreased more when mechanical plaque control was supplemented with the use of RV-HPβCD spray (*p* < 0.001), irrespective of toothbrushing behavior. No differences were observed in the control group between 2–3- and 4–5-year-old children. When considering clinical changes within each group, statistically significant improvement in FMPS was observed between baseline and 2 weeks and between 2 and 4 weeks in all groups. Conversely, in the test group, the oral spray application was more effective in reducing FMBS in the 4–5-year-old children between the 2- and 4-week examinations than in children aged 2 to 3 years. The same trend was observed in the control group but between baseline and 2 weeks.

**Figure 2:**
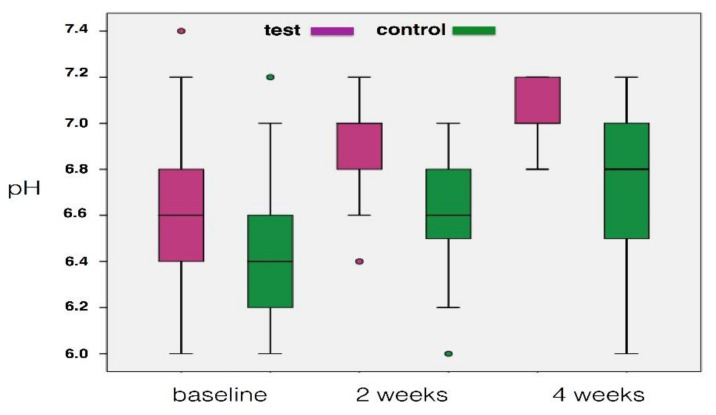
Changes of pH changes during the 4-week study period (box represents median, 25% and 75% percentiles, whiskers represent data within 10% and 90% percentiles).

As far as salivary parameters were concerned (Figure 2), pH value significantly increased in the control group between baseline and 2-week examination (*p* = 0.004), and then remained nearly unchanged. In contrast, the combined mechanical and chemical plaque removal was effective in progressively increasing pH value, which reached the neutrality between baseline and 2 weeks (*p* = 0.010) and further slightly increased from the 2- to 4-week examination (*p* = 0.031).

**Figure 3:**
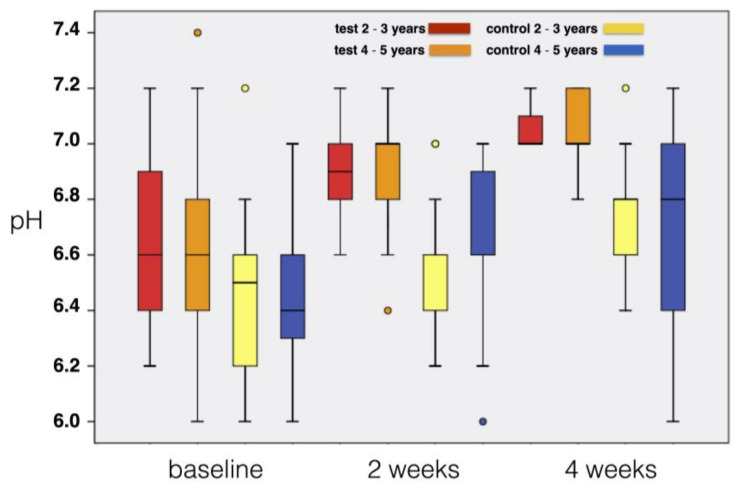
Changes of pH during the 4-week study according to the age of the children and the treatment group (box represents median, 25% and 75% percentiles, whiskers represent data within 10% and 90% percentiles).

The observed changes were not dependent on the age of the children: Higher pH increase was obtained in the test group as compared to the control group irrespective of the toothbrushing habits at both 2 and 4 weeks (Figure 3).

### 3.3. Adverse Effects and Patient-Centered Outcomes

No adverse reactions affected the hard and/or soft tissues during the 4-week study period. The 2- and 4-week intraoral examinations found no tissue changes and no teeth discoloration using the RV-HPβCD spray.

All parents reported no difficulty in using the spray and all the children tolerated the agent well. The taste of the spray was well accepted by all of them, regardless of the age group. Based on the emoticon scale, all children rated the spray as good (smile face).

## 4. Discussion

Plaque-induced gingivitis and dental decay are a challenge problem in preschool children who often show inadequate quality of plaque control. In case of poor oral hygiene, primary teeth are more prone to develop carious lesions than permanent teeth due to difference in enamel structure [28], with peak prevalence at 2–5 years. Therefore, effective plaque control should be a demanding objective. The aim of the present study was to investigate whether the addition of RV-HPβCD oral spray to oral hygiene procedures reduces plaque and gingivitis in children 2–5 years old, due to its potential for host modulation and antimicrobial activity [10]. RV alone exerts antibacterial effect against oral microbial pathogens involved in periodontal disease and dental caries, such as *Porphyromonas gingivalis*, *Aggregatibacter actinomycetemcomitans* [29,30] and *Streptococcus mutans* [31].

Here, we investigated the effect of RV nanoencapsulated with HPβCD (to improve its efficacy) on the existing plaque during the first two experimental weeks and on the rate of de novo biofilm accumulation on plaque-free teeth during the last two weeks of the study. The RV-HPβCD oral spray had a statistically and clinically significant effect on the amount of plaque after two and four weeks of daily use, once per day, and twice as effective as toothbrushing alone. FMPS decreased by 38.0% in the test group compared to 19.1% in the control group at two-week examination and FMPS amounted to 9.8% versus 25.8%, two weeks after the polishing session. The trend of FMBS paralleled that observed for FMPS values. This could be due to the remarkable pleiotropic activities (anti-inflammatory, anti-bacterial, anti-oxidant) of RV in the oral cavity [10]. It efficiently inhibits, not only the growth of Gram-negative periodontal pathogens [29,30], but also causes a decrease in oxidative burst capacity of neutrophils and in pro-inflammatory cytokines produced in the gingival tissues [11,13].

The current findings further support the utility of chemical plaque control in addition to daily oral hygiene measures in childhood. Unfortunately, it is difficult to find products suitable for pediatric patients. Chlorhexidine (CHX) is considered as the “gold standard” of oral antiseptics for its antimicrobial activity, low toxicity and high substantivity [32]. Nevertheless, CHX can lead to numerous side effects, such as the pigmentation of the dental surfaces and the mucous membranes, burning sensation, and alteration of taste resulting in a strong negative impact on patient’s compliance [33]. For all these reasons, CHX is not recommended for children younger than six years [34], who are not able to rinse and split without swallowing the mouthwash, with the risk of possible side effects due to the ingestion of the product.

Therefore, a mouthwash containing a natural compound in a spray formulation seems to be a good choice: Oral spray formulation has been recommended for physically and mentally challenged patients and institutionalized elders, but it could represent an interesting possibility for healthy preschool children, too [8].

It is meaningful that a significant clinical improvement was also observed after use of daily toothbrushing only, and this underlines the importance of oral health education either for children or parents in adopting appropriate brushing technique and frequency. It is evident that at least a temporary change in oral hygiene habits occurred, but repeated motivation sessions are needed to maintain improved gingival conditions over a long time period [35,36]. As reported in the literature, often parents neglect the importance of primary teeth carious lesions [37] and leave them untreated so that children do not have a clear perception why they should regularly brush their teeth [38]. According to the Italian guidelines on Dental Prevention, parents should be directly involved in their children’s toothbrushing habits until three years of age, and afterwards, they should help children perform toothbrushing procedures until they acquire sufficient manual skills [21].

When data were stratified on the age of the enrolled children, the efficacy of the spray was found not dependent on the toothbrushing behavior. However, among children under three years old, the spray was effective in controlling gingival inflammation after four weeks of daily use. In the control group FMPS values were higher among children aged 2–3 years than in the 4–5 years group at both two-week and four-week examinations even if the difference did not reach statistical significance, probably due to the smaller number of children under three years of age.

Concerning changes in salivary pH, the test group experienced a more marked tendency to neutral values than that observed in the control group. Consistent with the abovementioned findings, there was no significant difference in this parameter within each subgroup based on child’s age and toothbrushing behavior. Although the literature reveals controversial results for the associations between salivary parameters and dental decay, the research reports a positive correlation between pH value, prevalence of caries and salivary counts for *Streptococcus mutans* [39,40,41], even if it should be considered that changes in pH could be due to both intrinsic factors (such as buffering capacity of saliva) and extrinsic ones (such as oral hygiene and lifestyle habits) [42]. Therefore, it is reasonable to assume that the effect of the oral spray may be due to the greater improvement in oral hygiene level in the test compared to the control group. Proper oral hygiene prevents the development of mature bacterial biofilm and increases the probability that microbiota remains acidogenic-bacteria free [43].

In order to monitor tooth staining and taste alteration as well as potential adverse effects on oral tissues as a result of RV daily use, intraoral examinations were performed during every visit. No patient experienced areas of staining on the anterior teeth after 4 weeks of using the spray and/or local effects. Parents reported that their children did not show resistance to the use of the product, due to the spray formulation so they did not have to discontinue its application, but they were willing to use it for longer periods.

Taste is regarded as the main feature determining acceptability of a liquid medicine in pediatric populations [44]. We used a face-rating score system to help children in evaluating their taste perception. All children scored positively the palatability of the oral spray. However, it should be taken in consideration that there is not a gold standard for taste perception in children before the age of six years and it is possible that their rating is influenced by the parental behavior [45].

A limitation of this study is not having used a placebo spray in the control group. The bias introduced by the placebo effect in clinical trials has been previously documented [46]; so it is not possible to rule out that the act itself of using or not using the spray may have had some effects on the outcomes. There were no deviations from the assigned intervention and all patients completed the trial. Furthermore, the present results were confirmed by other data reported in literature concerning the combination of mechanical oral hygiene plus placebo rinses [8]. Another limit is represented by the short duration of the investigation. However, since RV nanocomplexed with HPβCD was safe and well-accepted by parents and children, it can be envisaged its application beyond the time set by this study. Finally, a microbiologic analysis was not performed. However, it is reasonable to assume that the antibacterial effect of the RV spray can reduce the dental load of *Streptococcus mutans*. It is also important to consider that RV application can interfere with the ability of *Streptococcus mutans* to produce an acidic environment inhibiting its cariogenic virulence [31].

## 5. Conclusions

Oral sprays may have potential as an alternative delivery method in early childhood and natural molecules may be more appropriate to be used in these products than chemical compounds. An oral spray application with RV-HPβCD once a day resulted in marked decreases in dental plaque level and gingival inflammation, but also in neutral pH value, and this may be considered a promising new approach to improve oral health in preschool children. Long-term controlled studies on the effectiveness of RV oral spray are needed and encouraged.

## Figures and Tables

**Table 1:** Demographic characteristics of the study population (mean ± standard deviation or *n* [%]).

Variable	Test Group (*n* = 32)	Control Group (*n* = 32)	*p* Value Test vs. Control
Age (years)	3.9 ± 0.9	3.7 ± 1.0	0.426
Range	2–5	2–5	-
Females	15 (46.9)	17 (53.1)	0.617

**Table 2:** Changes in clinical variables (mean ± standard deviation) during the 4-week study period.

Variables	Group	Baseline	2 Weeks	Δ_0–2 weeks_	4 Weeks	Δ_0–4 weeks_
FMPS (%)	Test	60.9 ± 9.5 ^a^	22.9 ± 6.0 ^b^	38.0 ± 9.5	9.8 ± 2.9 ^b^	51.1 ± 10.5
	Control	57.6 ± 10.7 ^a^	38.5 ± 11.4 ^b^	19.1 ± 10.5	25.8 ± 7.3 ^b^	31.8 ± 12.4
Difference between groups		NS	<0.01		<0.001	
FMBS (%)	Test	27.8 ± 10.5 ^a^	9.6 ± 4.1 ^b^	20.2 ± 10.3	3.9 ± 2.7 ^b^	25.9 ± 10.6
	Control	31.6 ± 9.8 ^a^	19.8 ± 8.7 ^b^	11.8 ± 9.5	12.2 ± 4.4 ^b^	19.4 ± 8.9
Difference between groups		NS	<0.001		<0.001	

FMPS, Full-Mouth Plaque Score; FMBS, full-mouth bleeding score; NS, difference between groups not statistically significant (*p* > 0.05); ^a^ *p* < 0.001, *p* values represent changes among the three time points; ^b^ *p* < 0.001, *p* values represent longitudinal changes from baseline.

**Table 3:** Changes in clinical variables (mean ± standard deviation) during the four-week study period according to the age of the children and the treatment group.

Variable	Time	Test Group		Control Group		*p* Value			
		2–3 Years (*n* = 11) A	4–5 Years (*n* = 21) B	2–3 Years (*n* = 13) C	4–5 Years (*n* = 19) D	A vs. B	A vs. C	B vs. D	C vs. D
FMPS (%)	Baseline ^a^ (T_0_)	60.8 ± 9.0	61.0 ± 9.9	57.6 ± 12.1	58.7 ± 10.0	NS	NS	NS	NS
	2 weeks ^b^ (T_1_)	23.2 ± 5.8	22.8 ± 6.2	41.6 ± 12.5	36.5 ± 10.4	NS	<0.001	<0.001	NS
	*p* T_0_ vs. T_1_	<0.001	<0.001	0.002	0.001				
	4 weeks ^b^ (T_2_)	8.8 ± 3.2	10.2 ± 2.6	28.0 ± 5.2	24.4 ± 8.2	NS	<0.001	<0.001	NS
	*p* T_1_ vs. T_2_	0.001	<0.001	0.005	0.006				
FMBS (%)	Baseline ^a^ (T_0_)	32.3 ± 9.3	28.5 ± 11.1	32.4 ± 10.3	31.1 ± 9.7	NS	NS	NS	NS
	2 weeks ^b^ (T_1_)	9.4 ± 3.0	9.7 ± 4.5	21.8 ± 9.5	18.5 ± 8.1	NS	<0.001	0.001	NS
	*p* T_0_ vs. T_1_	< 0.001	0.002	NS	0.006				
	4 weeks ^b^ (T_2_)	4.8 ± 3.7	3.5 ± 1.8	12.7 ± 4.1	11.9 ± 4.6	NS	<0.001	<0.001	NS
	*p* T_1_ vs. T_2_	NS	0.001	0.018	0.002				

FMPS, Full-Mouth Plaque Score; FMBS, full-mouth bleeding score; NS, difference between groups not statistically significant (*p* > 0.05); ^a^ Difference among groups not statistically significant (*p* > 0.05); ^b^ Difference among groups statistically significant (*p* < 0.001).

## Data Availability

The data presented in this study are available upon request from the corresponding author.
